# Defined d-hexapeptides bind CUG repeats and rescue phenotypes of myotonic dystrophy myotubes in a *Drosophila* model of the disease

**DOI:** 10.1038/s41598-021-98866-0

**Published:** 2021-09-30

**Authors:** Anna Rapisarda, Ariadna Bargiela, Beatriz Llamusi, Isabel Pont, Roger Estrada-Tejedor, Enrique Garcia-España, Ruben Artero, Manuel Perez-Alonso

**Affiliations:** 1 Department of Genetics, University Institute for Biotechnology and Biomedicine (BIOTECMED), University of Valencia, Dr. Moliner, 50, 46100 Burjasot, Valencia, Spain; 2 Translational Genomics Group, INCLIVA Biomedical Research Institute, 46010 Valencia, Spain; 3 IQS School of Engineering, Universitat Ramon Llull, Barcelona, Spain; 4 Instituto de Ciencia Molecular (ICMol), Departamento de Química Inorgánica, C. Catedrático José Beltrán 2, Universidad de Valencia, 46980 Paterna, Spain; 5 Present Address: Arthex Biotech S.L. Catedrático Agustín Escardino 9, Parc Scientific University of Valencia, Paterna, Valencia, Spain

**Keywords:** Biotechnology, Drug discovery, Genetics, Molecular biology, Diseases

## Abstract

In Myotonic Dystrophy type 1 (DM1), a non-coding CTG repeats rare expansion disease; toxic double-stranded RNA hairpins sequester the RNA-binding proteins Muscleblind-like 1 and 2 (MBNL1 and 2) and trigger other DM1-related pathogenesis pathway defects. In this paper, we characterize four d-amino acid hexapeptides identified together with abp1, a peptide previously shown to stabilize CUG RNA in its single-stranded conformation. With the generalized sequence cpy(a/t)(q/w)e, these related peptides improved three MBNL-regulated exon inclusions in DM1-derived cells. Subsequent experiments showed that these compounds generally increased the relative expression of MBNL1 and its nuclear-cytoplasmic distribution, reduced hyperactivated autophagy, and increased the percentage of differentiated (Desmin-positive) cells in vitro. All peptides rescued atrophy of indirect flight muscles in a *Drosophila* model of the disease, and partially rescued muscle function according to climbing and flight tests. Investigation of their mechanism of action supports that all four compounds can bind to CUG repeats with slightly different association constant, but binding did not strongly influence the secondary structure of the toxic RNA in contrast to abp1. Finally, molecular modeling suggests a detailed view of the interactions of peptide-CUG RNA complexes useful in the chemical optimization of compounds.

## Introduction

Myotonic dystrophy type 1 (DM1; OMIM #160900) is a rare autosomal dominant disease with symptoms that typically affect the musculoskeletal system, with degenerative muscle atrophy and myotonia (or muscle hyperexcitability), heart conduction defects, and cognitive involvement, which combined with several other multisystemic alterations severely affect the life expectancy and quality of life of patients^1^. DM1 originates from an abnormal expansion of an unstable CTG trinucleotide repeat in the 3’-untranslated region of the *DM1 protein kinase* (*DMPK*) gene^2^. Expanded CTG repeats are transcribed but not translated and get retained in the cell nucleus where mutant transcripts accumulate, forming foci that sequester proteins of the Muscleblind-like (MBNL) family of alternative splicing regulators, among other molecular consequences^3^. The CUG expansions fold into metastable hairpin structures that facilitate the binding and sequestration of nuclear factors, among which MBNL1 is the most relevant in this work. Specifically, it has been shown that compound loss of *Mbnl1* and *Mbnl2* in mice reproduce several DM1 symptoms^4^. At the same time, Mbnl1 overexpression rescues DM1 phenotypes in a mouse model that expresses CTG repeats throughout the skeletal muscles^5,6^. Human MBNL1 proteins are tissue-specific CCCH zinc finger factors with crucial roles in the regulation of alternative splicing and alternative polyadenylation during development, in which it promotes a switch from fetal to adult patterns in a wide number of transcripts^7–9^. Although lack of MBNL1 function is one of the main molecular hallmarks of DM1 myopathy, many additional molecular contributors have been reported^10^. Hyperactivated GSKbeta and autophagy have been proposed to contribute to muscle atrophy in DM1 by stabilizing a repressive form of CELF1 alternative splicing regulator in the nucleus and downregulation of miR-7, a master regulator of autophagy, respectively^11–13^. Although most therapeutic strategies have focused on degrading the expanded CUG RNA or preventing MBNL sequestration by the toxic RNA, with small molecules or oligonucleotide-based approaches^14,15^, direct upregulation of endogenous MBNL1 levels is becoming accepted as a complementary approach^16–18^.

*Drosophila* is one of the experimentation animals used to model DM1 by expressing non-coding CTG repeat expansions to the insect muscles, brain, and heart to reproduce critical DM1 molecular defects and test candidate therapeutics^19–21^. We previously targeted the expression of 480 interrupted CTG repeats to the *Drosophila* mushroom bodies, which are a pair of brain structures in insects. This generates a semi-lethal phenotype at the pupal stage used to screen a positional scanning synthetic combinatorial library of d-amino-acid hexapeptides that identified 16 candidate peptides, of which Abp1 (ppyawe) was characterized in more detail^22^. Our current study addressed the characterization of the remaining 15 peptides in a secondary screen in a DM1-derived cell model of the disease and identified four closely related peptides that improved cell and *Drosophila* DM1 phenotypes by directly binding to the CUG RNA.

## Results

### Four related peptides rescue MBNL-dependent missplicing events in DM1 myotubes

**Figure 1:**
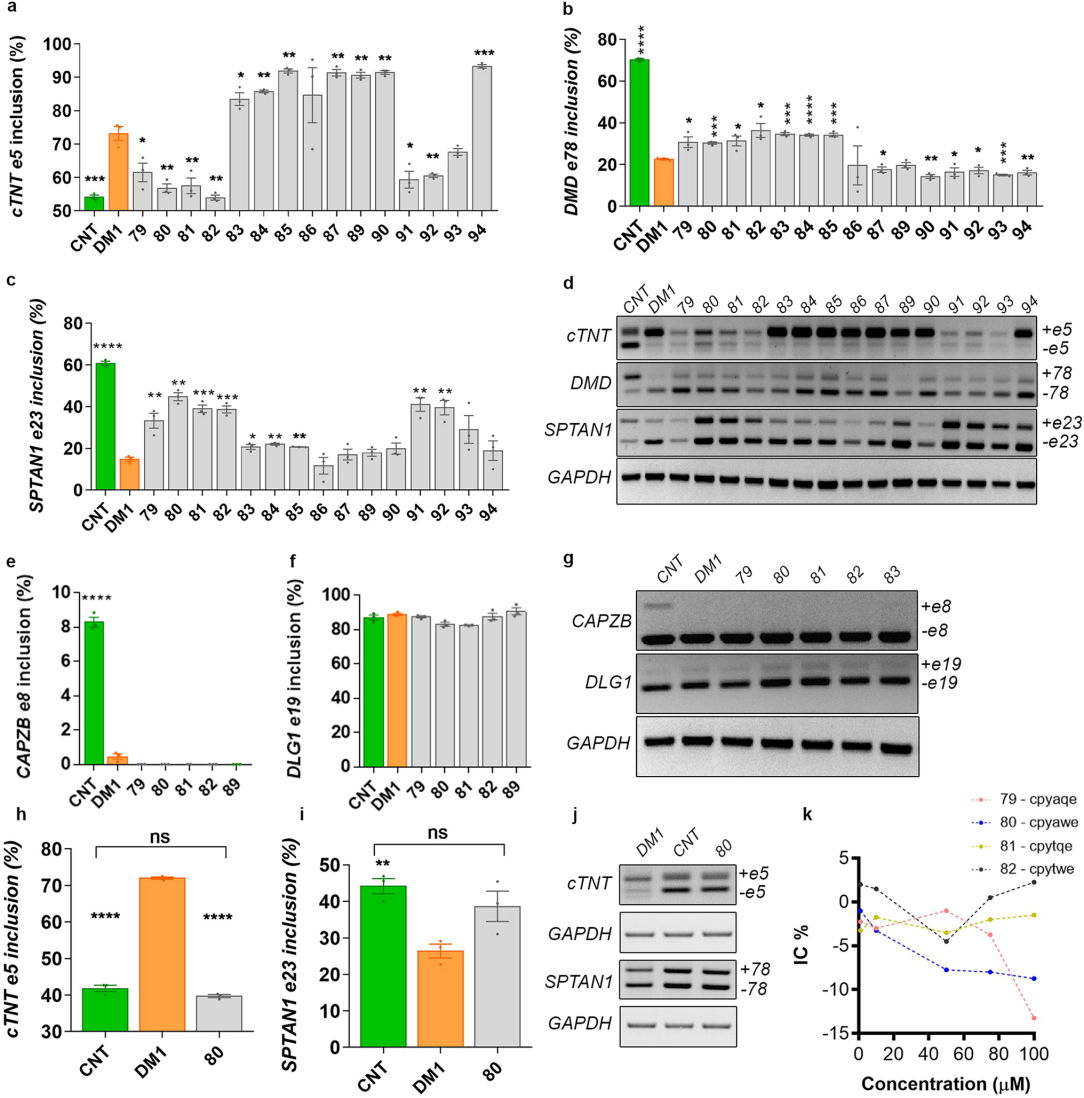
Peptides 79, 80, 81, and 82 rescued MBNL-dependent mis-splicing events in DM1 myotubes. Control (CNT, with no DMSO) and DM1 myotubes (96 h of differentiation, 3 biological replicates of each condition) were treated with 10 μM of the indicated peptides or DMSO (0.1%) for 48 h and the percentage of inclusion of cTNT exon 5 (**a**), DMD exon 78 (**b**), and SPTAN1 exon 23 (**c**) was determined. (**d**) Representative 2% agarose gels showing semiquantitative RT-PCR amplicons with or without the indicated exons and GAPDH internal control. None of the tested peptides induced changes in the splicing of the CAPZB gene, altered in DM1 and regulated by the CELF1 protein (**e**, **g**) or in the splicing of the DLG1 gene, which remains unchanged in DM1 (**f**, **g**). The inclusion of exon 5 of the cTNT gene, regulated by MBNL1, (**h**, **j**) and the inclusion of the exon 23 of the gene SPTAN1, regulated by MBNL2 (**i**, **j**), did not respond to peptide 80 in CNT myotubes, thus supporting the specificity of its activity. Cell growth inhibition assay by MTS method. Human CNT myotubes were transfected with increasing concentrations of the indicated peptides (4 biological replicates per condition) (**k**). *p < 0.05, **p < 0.01, ***p < 0.001, ****p < 0.0001, ns p > 0.05 according to Student’s t-test.

Immortalized human DM1 muscle cell lines display disease-associated molecular features such as nuclear RNA aggregates and alternative splicing defects^23^. DM1-related phenotypes can be used as readouts to screen candidate therapeutics in vitro for effects on RNA toxicity associated with the DM1 mutation. MBNL protein depletion explains most aberrant splicing patterns observed in DM1^24–26^. Thus, we established three splicing events typically altered in DM1 as screening criteria for the 15 d-amino-acid hexapeptides previously identified (Supplementary Table 1)^22^: the inclusion of exon 5 of *cardiac troponin T* gene (*cTNT*; Entrez ID: 7139) and the exclusion of exon 78 of the *dystrophin* gene (*DMD*; Entrez ID: 1756), both MBNL1-dependent and the exclusion of exon 23 of *spectrin alpha non-erythrocytic* gene (*SPTAN1*; Entrez ID: 6709), which is MBNL2-dependent^27^. Immortalized control and DM1 fibroblasts were transdifferentiated into myotubes for 48 h. After that, they were incubated two more days with 10 µM of each peptide dissolved in myotube differentiation medium (MDM). Semiquantitative RT-PCR evaluated the activity of the peptides on the missplicing events. Despite most peptides being able to improve inclusion of at least one of the alternative exons, only peptides cpyaqe (79), cpyawe (80), cpytqw (81), and cpytwe (82) rescued all of them in a statistically significant manner (Fig. 1a–d and Supplementary Fig. 1). Notably, the four peptides shared 4 out of 6 amino acids, and only the fourth and the fifth positions changed, generating the consensus sequence cpy(a/t)(q/w)e, which strongly suggests a structure–function relationship. Treatment with these peptides did not change the inclusion of exon 8 of the *CAPZB* gene, regulated by CELF1^28^, nor exon 19 of the *DLG1* gene, which remains unchanged in DM1 patients^29^, suggesting a specific effect on the regulatory factors MBNL1 and 2 in the disease (Fig. 1e–g). Finally, we tested whether such activity was (CUG)_exp_-specific or not; thus, we treated control cells with 10 µM of peptide 80 and quantified their activity on the alternative exons of *cTNT* and *SPTAN*. The peptide produced no significant change, suggesting that its activity depended on the presence of the mutation that causes DM1 (Fig. 1h–j). Peptides 79, 80, 81, and 82 were selected for further evaluation. We treated control myotubes (Fig. 1k) and fibroblasts (Supplementary Fig. 2) with peptides 79, 80, 81, and 82 at concentrations ranging from 0.1 to 100 µM and assayed their toxicity profile. We did not observe any toxic effect even at the highest evaluated concentration. Thus, we performed following experiments at 10 µM, a concentration at which compounds were not toxic for cells.

### Candidate peptides enhance MBNL expression and its normal distribution in the cell

**Figure 2:**
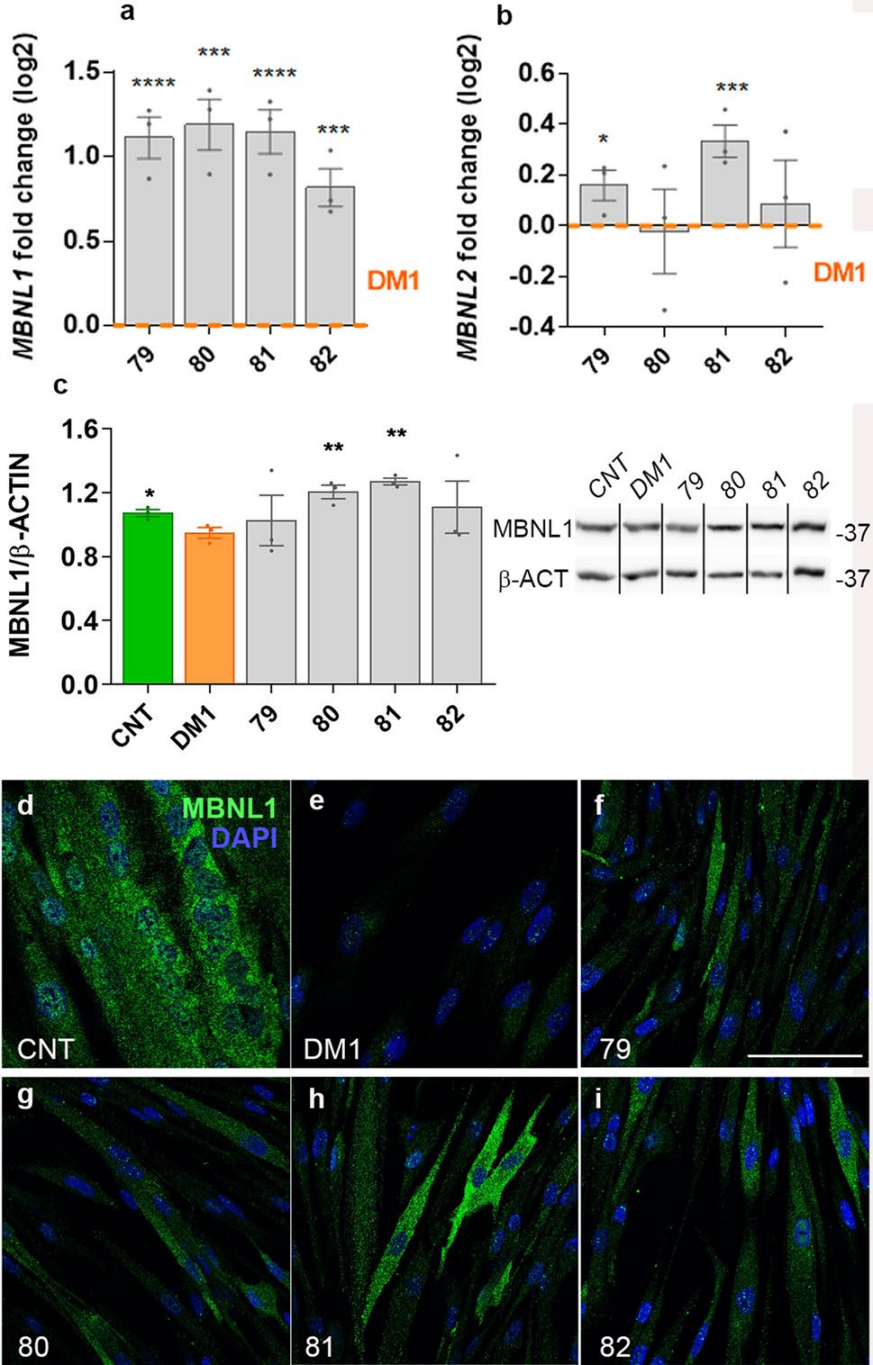
Candidate peptides enhanced the expression of MBNL proteins in a DM1 cell model. Quantification by RT-qPCR of relative expression of MBNL1 and MBNL2 transcripts in DM1 cells differentiated for four days and treated with the four candidate peptides (three biological replicates and three technical replicates per biological sample were performed). Levels were referenced to GAPDH as endogenous control (**a**, **b**). Western blot quantification relative to beta-Actin confirmed a significant increase in the levels of MBNL1 protein in the cells treated with the 80 and 81 peptides (3 biological replicates per condition). Black lines indicate those lanes cropped from different blots (**c**). The molecular weight marker band at 37 kDa is indicated to the right of the blot. Confocal microscopy micrographs of control (CNT, **d**; without DMSO) and DM1 myotubes (**e**–**i**) treated with vehicle (DMSO 0.1%; **e**) or the indicated peptides (**f**–**i**) stained for MBNL1 (green channel) and DAPI. All images were taken at the same settings. Note the general increase in the intensity of MBNL1 fluorescence in (**f**–**i**) panels. *p < 0.05, **p < 0.01, ***p < 0.001 according to Student’s t-test. Scale bar corresponds to 100 microns.

To understand what could trigger the rescue of the analyzed splicing events, we used cDNA from treated cells to perform quantitative PCR (qPCR) and detect any modification in the mRNA levels of *MBNL1* and *MBNL2*. We found that peptides 79, 80, 81, and 82 doubled *MBNL1* expression while peptides 79 and 81 slightly increased *MBNL2* mRNA amounts (Fig. 2a, b). This finding was encouraging as the increase in MBNL1 and 2 gene expression has been proposed as a valid strategy to improve the clinical outcome of DM1^5,6,16,18,19,30^. However, at the protein level, only cells treated with the peptides 80 and 81 showed a statistically significant increase in MBNL1 levels by western blot compared to untreated DM1 cells (Fig. 2c, Supplementary Fig. 3). Finally, since the subcellular localization of MBNL1 is altered in DM1^19,30^, we evaluated this phenotype using an anti-MBNL1 antibody. Immunofluorescence images confirmed that the MBNL1 signal was increased in the cytoplasm of the treated cells compared to the untreated and approached normal intensity and subcellular distribution (Fig. 2d–i). Taken together, these results indicate that the candidate peptides were able to target the upregulation of MBNL1. Therefore, we studied additional molecular phenotypes related to MBNL1 in the pathogenesis pathway.

### A 48-h treatment is sufficient to reduce the number of foci with no effect on the *DMPK* transcripts.

**Figure 3:**
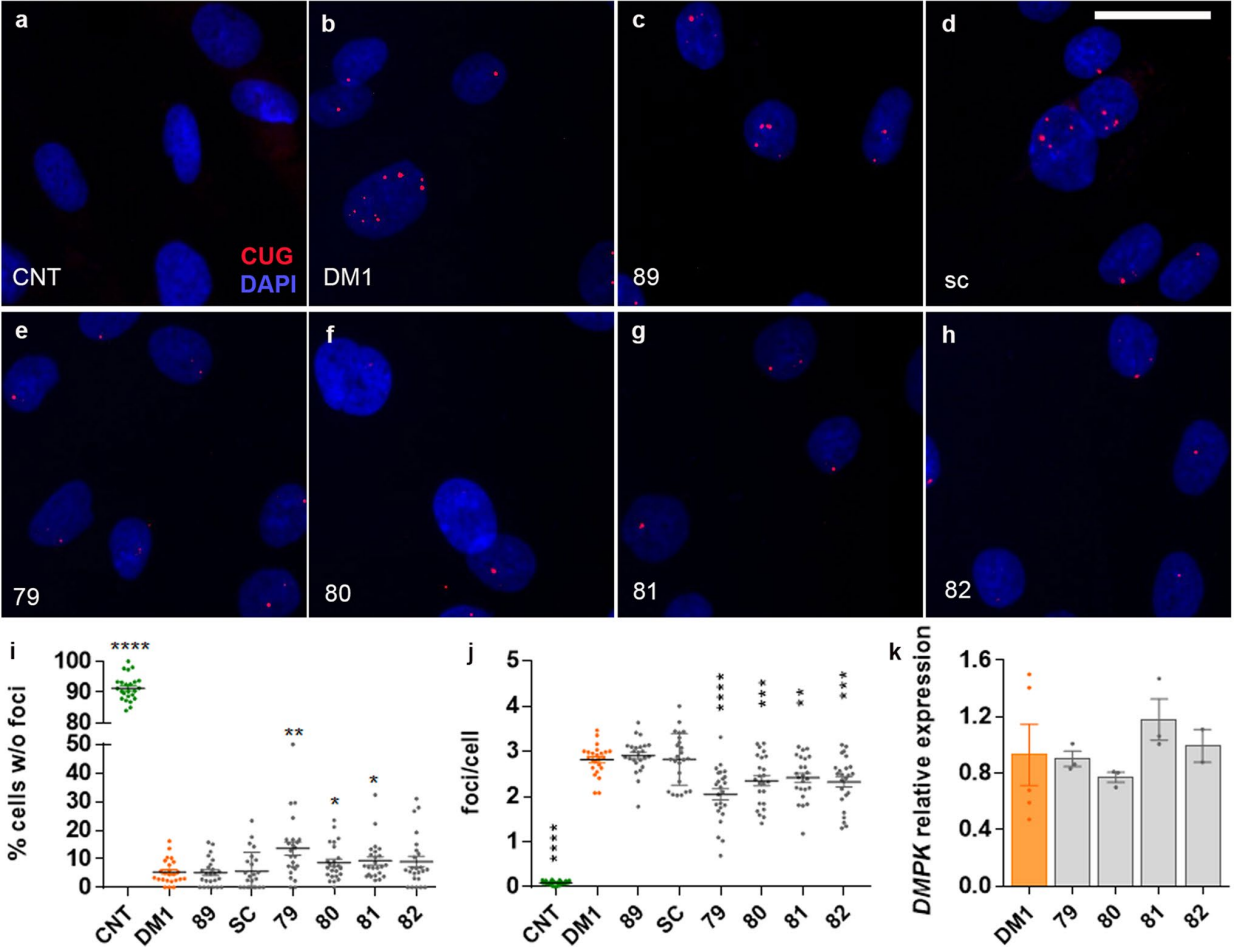
Candidate peptides reduced ribonuclear foci in DM1 cells. Representative micrographs of foci in control (CNT, **a**; without DMSO), DM1 (DMSO 0.1%, **b**), and DM1 cells treated with negative control (peptide 89 or scrambled peptide (SC); **c**, **d**) or candidate peptides (**e**–**h**) obtained with an IN Cell Analyzer high-content imaging system (three biological replicates and three technical replicates per biological sample were performed in each condition. Four different fields were analyzed in each sample). Accumulation of mutant transcripts was detected using fluorescent in situ hybridization (FISH) with a Cy3-labeled RNA probe (red dots). Nuclei were counterstained with Hoechst 33258 (blue). Quantification of the images revealed that peptides 79, 80, and 81 significantly increased the percentage of cells without foci (**i**) and reduced the number of foci per cell, in this case including peptide 82 (**j**). The observed reduction in mutant DMPK accumulation was not due to the repression of the DMPK gene expression itself, which was quantified by real-time PCR using primers against a non-repetitive sequence and was found not significantly different from DM1 controls (three biological replicates and three technical replicates per biological sample were performed; **k**). *p < 0.05, **p < 0.01, ***p < 0.001 according to Student’s t-test. Scale bar (**a**–**h**) measures 20 microns.

A prime mechanism to enhance functional MBNL1 levels in cells is to prevent its sequestering into ribonuclear foci by either blocking its binding to CUG repeats or changing the secondary structure of the toxic RNA so it is less prone to bind to the protein^15,22,31^. We quantified the number of foci per nucleus to shed light on this problem, using fluorescence in situ hybridization with an RNA probe (Cy3-(CAG)7-Cy3) and an IN Cell Analyzer High-Content Cellular Analysis System to acquire images (Fig. 3a–h). The treatment with each of the peptides produced a significant, although mild, change in the number of cells without foci, which increased, and in the percentage of foci per cell, which was significantly reduced (Fig. 3i, j). These changes occurred without altering the relative expression levels of *DMPK* transcripts (Fig. 3k), half of which carry the expanded CUG triplets, suggesting the possibility of a direct interaction of the peptides with the RNA, with or without a subsequent influence on its secondary structure.

### Treated DM1 myotubes reduce the differentiation delay

**Figure 4:**
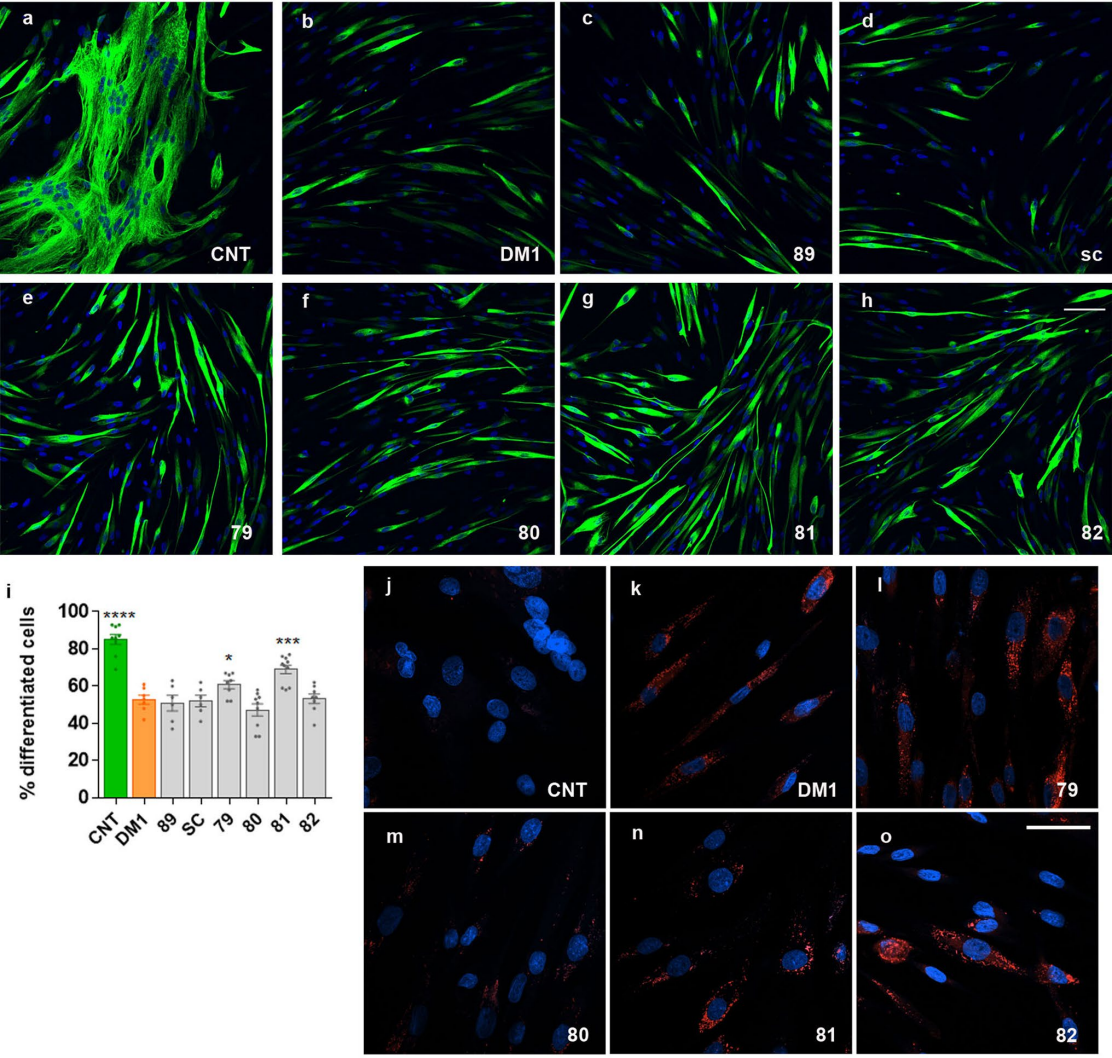
Differentiation delay and autophagy hyperactivation were rescued by peptides. Representative micrographs of control (CNT, **a**; without DMSO), DM1 (DMSO 0.1%; **b**) and DM1 cells treated with negative control (**c**, **d**) or candidate peptides (**e**–**h**) stained for Desmin (green signal) as a marker of myogenic differentiation. Cells differentiated in DMEM for seven days and were treated with 10 µM of the indicated peptides. Quantification of the number of Desmin-positive cells relative to the total number of cells (**i**) revealed a significant increase in myogenic cells when treated with peptides 79 and 81 (counting over 250 nuclei from randomly chosen Desmin-positive cells from 5–7 micrographs). Human myotubes stained with LysoTracker (red fluorescence; **j**–**o**). Autolysosomal labeling is observed in DM1 myotubes (DM1) but not in controls (CNT), denoting increased autophagy in DM1 cells. Cells treated with peptide 80 showed a general reduction of the signal from auto lysosomal vesicles while for cells treated with peptides 81 and 82, an increase in the number of cells without autophagic vesicles around the nucleus was observed. (**a**–**h**, **j**–**o**) Nuclei were counterstained with Hoechst 33258 (blue). Three independent experiments were carried out. *p < 0.05, **p < 0.01, ***p < 0.001 according to Student’s t-test. Scale bar corresponds to 100 (**a**–**h**) and 40 microns (**j**–**o**).

Symptoms of myotonia, muscle weakness, and muscular atrophy are the main features of DM1^1^, and the molecular contributions to these symptoms are numerous^10^. One of them is a delay in the process of muscle differentiation, which can be quantified as a reduction in the fusion index after the induction of the fibroblasts to myotubes transdifferentiation^32,33^. After incubating with MDM both the control and DM1 fibroblasts for four and seven days and treatment with the peptides for 48 h, we carried out immunofluorescence with an anti-Desmin antibody and quantified Desmin-positive (differentiated) cells. While the percentage of terminally differentiated cells remained unchanged after four days in MDM medium, after seven days the percentage of Desmin-positive DM1 cells increased significantly upon treatment with peptides 79 and 81 and remained unchanged in the presence of a scrambled control peptide (Fig. 4a–i). The fusion index, however, did not significantly increase (Supplementary Fig. 4).

Another molecular mechanism contributing to muscle atrophy in DM1 is the activation of autophagy^12,13,34^. To check the autophagy status in DM1 cells after peptide treatments, we used the lysotracker reagent, which stains acidic lysosomes^35^. First, we confirmed that the level of autophagy of the diseased cells was considerably higher than that of healthy cells (Fig. 4j, k). Furthermore, a qualitative analysis of the autophagic pathway by LysoTracker staining suggested that the treatment with the peptide 80 caused reduction in the signal associated with the lysosomal vesicles, indicating a recovery of the normal autophagy levels. Along the same lines, peptides 81 and 82 increased the number of cells devoid of autophagic vesicles around the nucleus (Fig. 4j–o). Thus, these observations indicate a general reduction in the autophagy pathway, which has previously been shown to contribute to muscle atrophy in *Drosophila* and human cells in vitro^12,13,34^.

### Candidate peptides rescue muscle atrophy of a *Drosophila* model of the disease

**Figure 5:**
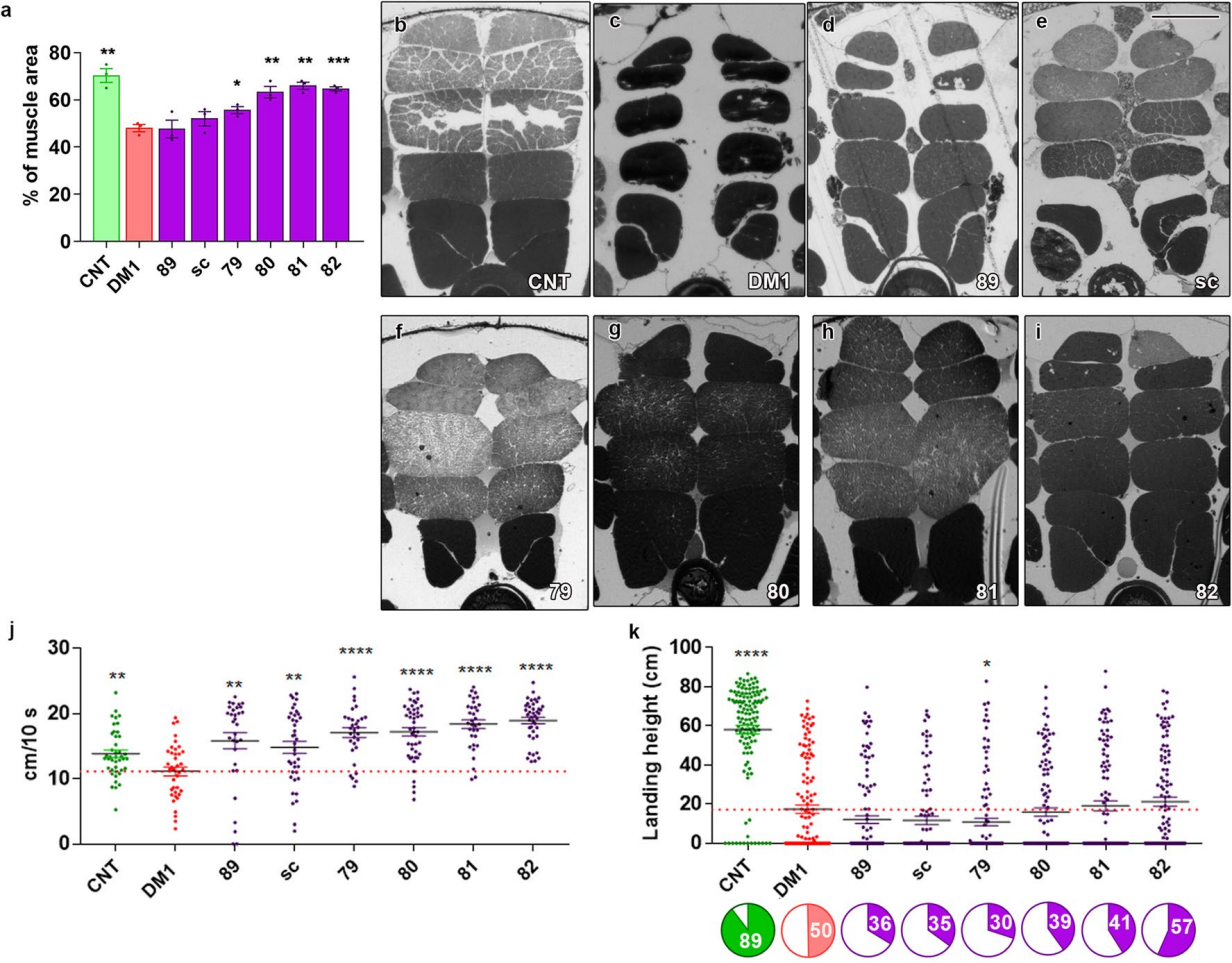
Candidate peptides rescued muscle atrophy in a Drosophila DM1 model. Quantification of indirect flight muscle (IFM) cross-sectional area (**a**) of control (CNT), DM1 flies taking vehicle (DMSO) and DM1 flies taking negative controls (89 or sc) or the indicated candidate peptides. All four candidate peptides increased the mean muscle area relative to DMSO-treated flies (6 flies and 6 micrographs per condition were analyzed). (**b**–**i**) Representative bright-field microscope images of transversal sections of resin-embedded adult IFM of Mhc-Gal4 UAS-i(CTG)480 (Rec2) heterozygous flies treated with DMSO (0.01%) or with peptides 79–82 (10 μM) that were used to generate the data shown in (**a**). Muscle recovery at the histological level leads to functional improvements. The climbing assay (n = 30; **j**) showed a significant increase in the speed of the treated flies compared to the untreated ones, calculated as the distance traveled in 10 s. In the flight assay (n = 100; **k**), the average value of the landing height did not significantly improve, but there was a clear increase in the percentage of flies that were able to fly (colored sectors in pie charts underneath the graph), especially for flies treated with peptide 82. *p < 0.05, **p < 0.01, ***p < 0.001 according to Student’s t-test. Scale bar for (**b**)–(**i**) panels measures 100 microns.

Rescue of two molecular phenotypes related to muscle atrophy in the cell model, namely delayed differentiation and hyperactivated autophagy, prompted us to verify if these peptides were also active in vivo in a muscle phenotype in *Drosophila*. In this model, the expression of toxic CUG repeats is controlled by the myosin heavy chain promoter and reproduces muscle phenotypes observed in humans^13,36,37^. After feeding the DM1 flies with standard food supplemented with the indicated peptides at a final concentration of 10 µM or DMSO as a control, we embedded the thorax of the flies to obtain cross-sections of indirect flight muscles. The quantification of the muscle area from these images showed a marked improvement in the atrophic phenotype in peptide-treated flies, bringing the muscle area to values very close to those observed in control flies (Fig. 5a–i). Concomitant to muscle atrophy, model flies have reduced locomotor abilities, which in flies can be assessed through climbing, taking advantage of *Drosophila’s* negative geotropism, and flight assays. First, we used 30 male flies for the climbing experiment to measure the height climbed by the flies in a given time. The results revealed a significant effect of the scrambled peptide, suggesting an unspecific effect of the compounds. However, comparing values obtained for flies fed with food supplemented with peptide 79, 80, 81 or 82 with the scrambled version we observed a significant increase in the speed of the flies treated with peptides 80, 81, and 82 (Fig. 5j). It is worthy of mentioning that climbing ability achieved by flies treated with the peptides was significantly higher than values obtained for control flies (p < 0.0008 in all cases). No significant differences were observed when comparing flies treated with control peptides and control flies. In flight tests, while there were no significant increases in the height of the landing distance (indicative of better flight capabilities), we found an increase in the percentage of flies capable of flying, especially in the case of peptide 82 treatment, where it reached statistical significance and almost doubled the value observed in control-treated DM1 flies (p = 0.0044, Fisher’s exact test; Fig. 5k). In conclusion, the increase in the number of flies showing the ability to fly indicates a partial rescue of the *Drosophila* muscle function consistent with the increase in the IFM muscle area.

### The secondary structure of (CUG)_23_ RNA remains unchanged after candidate peptides binding

**Figure 6:**
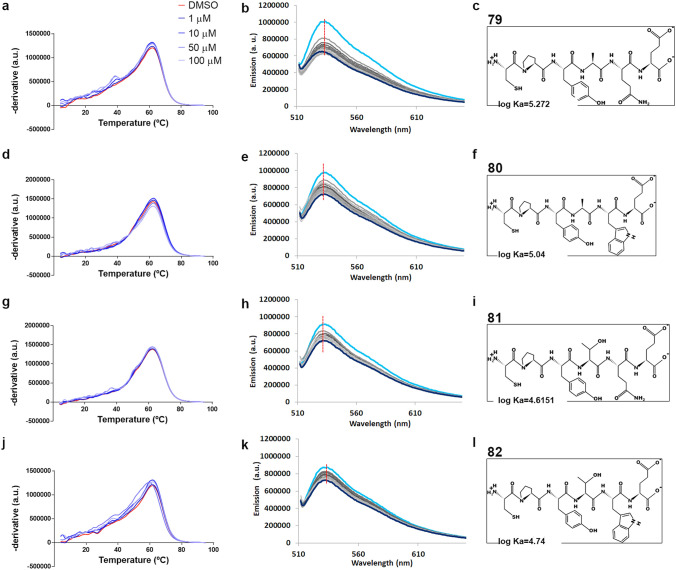
Candidate peptides interact with CUG RNA without affecting thermodynamic stability. The graphs show the first derivatives of RiboGreen fluorescence (DSF assay) versus temperature (**a**, **d**, **g**, **j**; 4 technical replicates per condition). and Thiazole orange fluorescence titration experiments (**b**, **e**, **h**, **k**; FID assay) for peptides 79 (**a**–**c**), 80 (**d**–**f**), 81 (**g**–**i**) and 82 (**j**–**l**). Experiment was performed per duplicate. DMSO 0.1% was used as a negative control. In Thiazole orange titrations, the intensity of the fluorescence progressively decreased with increasingly higher peptide concentrations (concentrations ranged from 0.03 up to 4 µM). The association constants (Ka) were calculated from FID assay data, which indicate that peptide 79 interacts with the (CUG)_23_ RNA probe with the highest affinity, although differences in binding affinity were low among all four peptides. DSF data also supports the binding of peptides to the RNA probe but did not detect significant changes in its secondary structure.

We used a Differential Scanning Fluorimetry (DSF) assay to monitor CUG RNA thermodynamics in the presence of increasing concentrations of candidate peptides. DSF is technique used to study the effect of compounds on RNA stability as RNA undertakes structural conversions upon thermal unfolding^38^. When RNA changes its structure the single stranded form increases the available binding sites for RiboGreen dye. We represented the first derivatives of normalized fluorescence of RiboGreen with an RNA probe containing 23 repeats of CUG versus temperature in the presence of concentrations of each hexapeptide ranging from 1 to 100 µM (Fig. 6a, d, g, j). The titration with increasing concentrations of peptides 79, 80, and 81 only changed the height of the peak indicating interference in the intrinsic folding properties of the RNA probe rather than stabilization or destabilization of the RNA hairpins. Peptide 82, however, did slightly shift the curve peak towards lower temperatures, which meant that the interaction between the hexapeptide and the probe does not stabilize the single strain RNA conformation, which is in striking contrast with the proposed destabilization of CUG RNA by abp1^22^. Taken together, the DSF experiments strongly support that the candidate peptides, at least 79, 80, and 81, do not significantly modify the secondary structure of the CUG RNA.

### Candidate peptides interact with CUG RNA with similar affinities

A fluorescent indicator displacement (FID) assay was used to investigate the nature of the interaction between the candidate peptides and the toxic RNA. Thiazole orange (TO) is an asymmetric cyanine intercalator with little fluorescence when free in an aqueous solution but strong emission when forming complexes of different nature with nucleic acids. These characteristics can be exploited to study changes in the interaction between the dye and the nucleic acid of interest in response to external factors^39,40^. Specifically, the fluorescent reporter interacted with the (CUG)_23_ RNA probe resulting in fluorescent emission. By adding the peptide, it was possible to displace the TO, causing its fluorescence to decrease. In this way, by analyzing the fluorescence emission at different RNA-peptide ratios, the value of the peptide's association constant for the CUG sequence could be indirectly calculated. For all peptides, a progressive reduction in fluorescence was observed in response to increasing concentrations of each peptide (Fig. 6b, e, h, k); in the case of peptide 79 (k_log_ = 5.273 ± 0.007), the greatest decrease in fluorescence was observed, followed by peptide 80 (k_log_ = 5.04 ± 0.01) and the smallest change was observed with peptides 82 and 81 (respectively k_log_ = 4.74 ± 0.02 and k_log_ = 4.65 ± 0.02) (Fig. 6c, f, i, l). However, it should be noted that in none of the cases did the level of fluorescence observed reach the characteristic values of a single-stranded RNA. This indicates that, although there was a clear interaction between the peptides and the RNA molecule, peptides did not interfere with the stability of the secondary structure of CUG RNA, which was consistent with the data generated by the DSF assay.

In parallel with the above experiments, we used the peptide showing the highest affinity for CUG repeats to investigate a potential direct interaction with MBNL1 proteins as an alternative mechanism of action since it might similarly prevent sequestration by the repeats. Double immunostaining with biotin-labeled peptide 79 revealed it accumulated in the cytoplasm of DM1 myotubes, mainly in the perinuclear area, but no significant overlap was found with the MBNL1 protein signal (Supplementary Fig. 5). Thus, candidate peptides do not seem to interact physically with MBNL proteins, at least peptide 79.

### Study of the interaction mechanism by molecular docking

**Figure 7:**
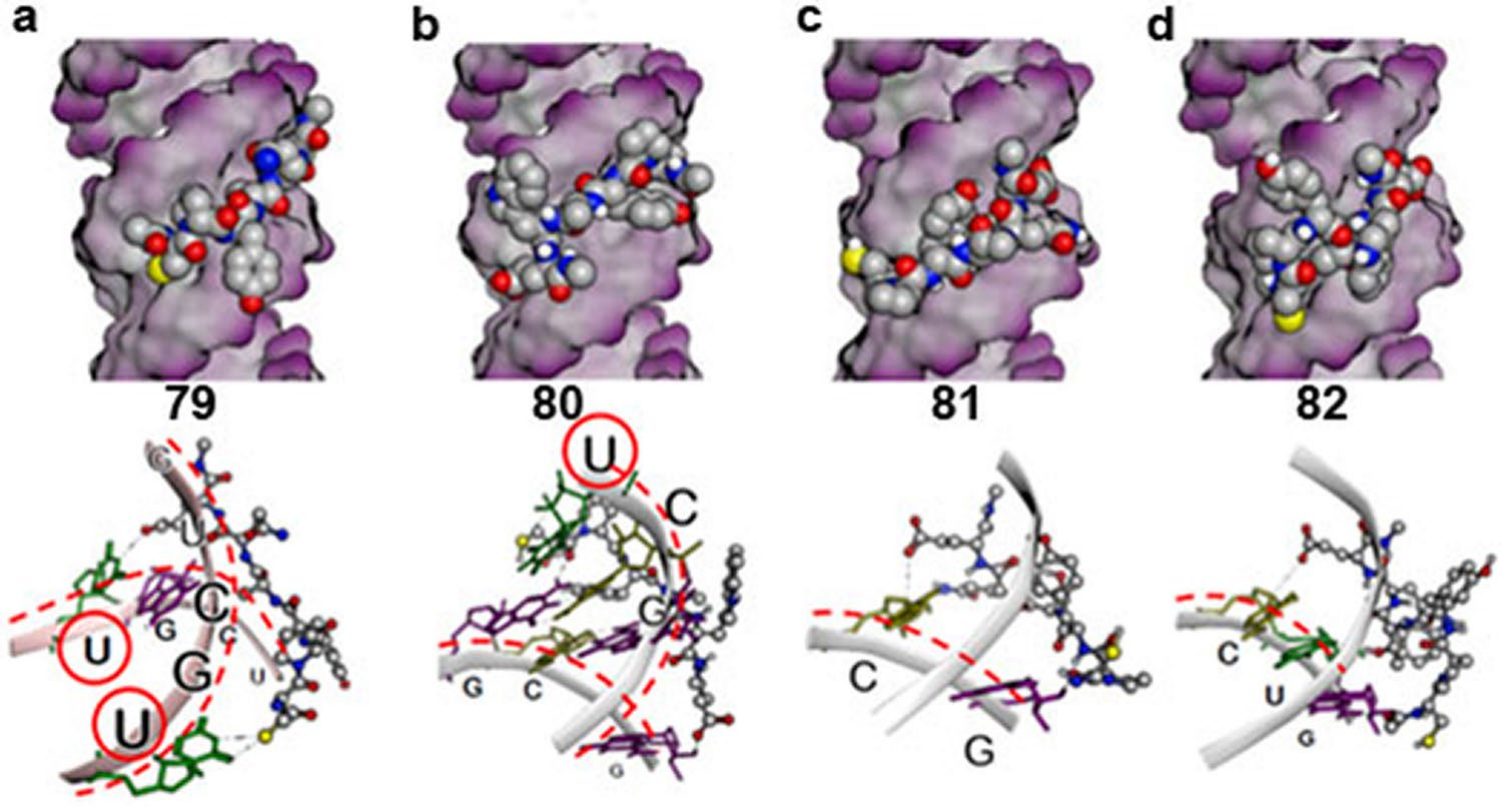
Modeling of the proposed interaction between the candidate peptides and the CUG RNA. Representation of the results obtained by means of a flexible docking directed to the area of the RNA exposed to the solvent. Two different binding mechanisms are proposed: peptides 79 (**a**) and 80 (**b**) might interact with the double-stranded RNA (red lines), recognizing the indicated uracils (red circles); peptides 81 (**c**) and 82 (**d**) might interact with one-stranded RNA (red line). Molecular representations included were generated using MOE 2019.01 software (Chemical Computing Group, https://www.chemcomp.com).

In molecular modeling, docking is a method that predicts the preferred orientation of one molecule to a second when they bind together to form a stable complex. Multiple docking studies were performed using Autodock VINA and Molecular Operating Environment (MOE) software to assess the preferred binding mechanism between hexapeptides and CUG repeats. Next, the results obtained with blind docking and guided docking techniques were compared; in the latter case, tests were carried out keeping the RNA rigid or admitting certain flexibility. The final docking protocol was validated by correlating the binding affinities predicted by docking (score) and FID results. According to the analysis, peptides 79 and 80 showed the most remarkable tendency to interact in the exposed part of the RNA (Fig. 7a, b). More in detail, peptide 79 was the only d-hexapeptide capable of recognizing two uracils of the two RNA chains by means of the two terminal amino acid residues. This observation could explain that it had the highest RNA binding association constant. Hexapeptide 80 would interact with the two strands of RNA but showing only the recognition of one uracil. According to the results, Trp would not be favorably available for interaction with the RNA backbone. This result is in agreement with the experimental data in which the presence of Trp did not contribute to the increase in the RNA binding constant (cpya**q**e > cpya**w**e, cpyt**q**e ~ cpyt**w**e). Although this conclusion could be due to a limitation of the simulation protocol used, which only contemplates slight flexibility of the RNA, it can be inferred that Trp would not interact by ππ-stacking (attractive and non-covalent interactions between aromatic rings). As for the two peptides with the lowest experimental interaction energy, it was surprising that the binding mechanism obtained, both for peptides 81 and 82, only showed interaction with one strand of RNA (Fig. 7c, d). Of the two, 82 could interact with three consecutive nucleotides, providing a slight additional stabilization.

## Discussion

In this paper, we report identifying four related peptides, with the sequence cpy(a/t)(q/w)e, that improved splicing and differentiation phenotypes in DM1-derived cells and reduced ribonuclear foci number through the direct binding to the repeats. Muscle area and functional defects in a *Drosophila* model also improved upon oral administration to the flies. We show that the candidate peptides bind CUG repeats with similar affinity but do not impinge on the secondary structure of the toxic RNA and propose a detailed mechanism of potential interactions between the peptides and the repeats. Although the peptides behaved similarly in many experimental conditions; all four peptides shared (1) a very similar sequence; (2) a similar ability to rescue missplicing in patient-derived cells, (3) the capacity to reduce foci number per cell, (4) the ability to improve histological and functional phenotypes in vivo, and (4) bound CUG RNA with a similar affinity and mechanism in in vitro assays, they also showed differences for example, in terms of doses at which they may show one biological activity or another, and potential mechanisms of action. Although additional experiments should be performed to account for the complexity of the therapeutic mechanism of each of the peptides there are some aspects that could help to shed light on the differences observed. The explanation for these differences could lie in the sequence variations, which are responsible for molecular structural changes. Minimal structural changes can significantly interfere in the molecular interaction between peptides and their targets^41^. Despite some unspecific effects by the scrambled peptide in climbing assays, the relevance of the sequence is clearly demonstrated in *Drosophila* muscle area determinations and in cell experiments in which SC peptide did not affect cross-sectional muscle area (Fig. 5a), the number of foci per cell, percentage of cells without foci (Fig. 3) or cell differentiation (Fig. 4).

**Figure 8:**
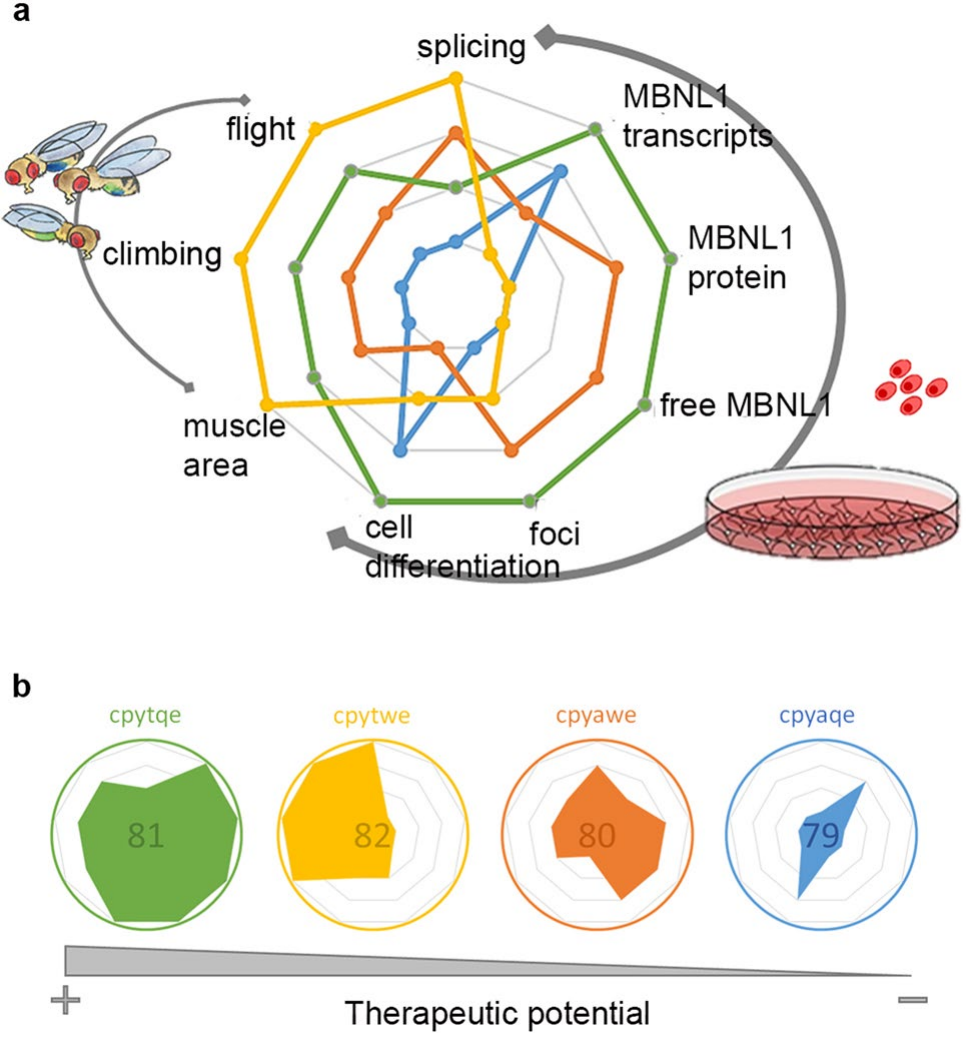
Overall assessment of candidate peptides. Rescues obtained with each peptide (according to the color code shown in **b**) were converted into a semiquantitative score that reflected the quality of rescue achieved in each of the experimental systems represented (Drosophila and cell model). Scores were inserted into a spider chart, where the internal most polygon indicates no rescue and the outermost indicates full rescue (**a**). The four peptides were sorted according to their therapeutic potential in decreasing order from left to right (**b**). Created with BioRender.com.

Peptide specificities are best illustrated as a spider graph, in which nine DM1-related parameters are simultaneously represented for each of the peptides using a semiquantitative scale that ranks peptides in each of these parameters (Fig. 8a). Thus, for example, while in terms of foci reduction peptide 81 shows the most activity, followed by 80, 82, and 79, for cell differentiation the rank was peptide 81 > 79 > 82 > 80. Indeed, since the higher the score, the better the rescue, we can integrate the area of the polynomial defined by each peptide to rank the overall therapeutic potential of peptides as 81 > 82 > 80 > 79 (Fig. 8b). These images also help illustrate that peptide 81 exhibited the best corrective capacity in DM1 patient cells, while peptide 82 was more effective in the *Drosophila* disease model.

Thanks to their intrinsic property of high specificity for a target biomolecule and the use of strategies to improve stability and uptake, and to reduce toxicity, several non-natural peptides have already reached medical use^42^. Indeed, once identified, peptides with promising anti-DM1 potential can be further improved using various strategies to develop a lead compound suitable for clinical studies^42–44^. Among the four candidate peptides, 81 is regarded as the most promising since it had the highest human cell activity and significant positive effects in *Drosophila*. The identified peptides are also very short, which in general terms can be expected to enhance distribution among biological tissues. In the hit-to-lead process, some well-established strategies could improve the pharmacological characteristics of the peptides. For example, various studies have shown that the bioactive peptide conjugation with a cell-penetrating peptide considerably enhanced its activity^45^. This is particularly important for a peptide that must reach the muscles. Therefore, it would be of interest to conjugate our candidate peptides with the TAT fragment (^48^GRKKRRQRRR^57^) or with a poly-Arginine peptide (Arg_5-8_), either directly or through a spacer linker^46^. Other successful strategies are the conversion of the peptide with the desired biological activity into a peptoid, in which the side chain is connected to the nitrogen of the peptide backbone, instead of the α-carbon as in peptides, and other types of peptidomimetics, to improve stability and cellular uptake^47^.

Our molecular modeling study offers a detailed view of how peptides 79–81 might be interacting with CUG repeats. One observation is that, given the known flexibility of the RNA under study, it is not surprising that the observed interactions may show fluctuations. Even so, the results indicate that the two interactions established by peptide 81 with C and G are more labile than the three presented by peptide 82, a fact that could explain its slightly higher binding constant. It is also observed that peptide 80 maintains its interaction with RNA in a significant way, although Trp is not capable of establishing a permanent interaction. Similarly, the interactions initially defined by peptide 79 with consecutive CUG uracils are labile but are recovered during the simulation. It is finally interesting to remark that peptides 79–81 start with a cysteine (c) and end with a glutamic acid (e), which is a structural feature conserved within the zinc fingers 2 and 4 of MBNL1, 2 and 3, and zinc finger 1 of MBNL2 and also show other coincidences with conserved amino acids within zinc fingers of these proteins in positions 4 (a) and 5 (q) (Supplementary Fig. 6). Interestingly, molecular modeling predicts the terminal residues of peptide 79 to interact with two U in the double-stranded CUG RNA (Fig. 7a).

In brief, we propose that 79–81 peptides bind CUG repeats and prevent MBNL1 depletion with little or no change in the secondary structure of the CUG RNA. On the basis of these results, it cannot be ruled out that the peptides interact with RNA sequences other than (CUG)_exp_. However, it is to be expected that the interaction is specific since treatment of healthy control cells with peptide 80 (Fig. 1 h, j) had no effect on the analyzed phenotypes while a significant improvement was detected when the peptide was added to DM1 muscle cells. Enhanced MBNL1 levels and close-to-normal distribution in the cell brings about cell model rescues at the molecular and cytological levels. We hypothesize that peptides are contributing to rescue this phenotype by two different pathways. Specifically, peptides treatment may promote increased MBNL1 (RNA and protein), and they may also stimulate MBNL1 release from ribonuclear foci by the interaction with the RNA, as their number was significantly reduced with the four peptides tested (Fig. 3). Indeed, data qualitatively showed that peptides impinge autophagic activity (Fig. 4) and we have previously demonstrated that the inhibition of this pathologically hyperactivated pathway in DM1 led, among other consequences, to increased levels of MBNL. Therefore, we propose that reduced autophagy induced by peptides treatment may finally lead to increased MBNL1^19^ as it reduces its degradation in the autophagolysosomes.

Despite the close similarity of 79–81 peptides with the previously identified abp1^22^, cpy(a/t)(q/w)e and ppyawe, respectively, the mechanism abp1 used to prevent sequestration was the stabilization of CUG RNA in its single-stranded conformation compared to an apparent sterical hindrance by 79–81. Together with abp1, these peptides offer relevant substrates for more focused medicinal chemistry studies towards developing therapies for myotonic dystrophy.

## Methods

### Hexapeptides used

d-amino acid hexapeptides used in this work (Supplementary Table 1) were purchased from GenScript (purity > 98%) and were dissolved in 100% DMSO as stock solutions and stored at room temperature until use.

### *Drosophila* methods

For the evaluation of compounds*,* we used a previously described recombinant *Drosophila* line in which a *UAS-(iCTG)*_*480*_ transgene (480 interrupted CTG repeats) was combined with the *Myosin heavy chain (Mhc)-Gal4* driver for continuous expression of the toxic RNA in the fly muscles, hereafter referred to as Rec-2^36^. For the crosses, 20 *w*^*1118*^ females and 10 Rec2 males were crossed and 12 females of the offspring were hand-collected and transferred to a tube containing 3 ml of regular *Drosophila* medium supplemented with the indicated peptides at a final concentration of 10 μM in 0.01% DMSO, or 0.01% of DMSO as a control. Flies were moved to a tube with fresh food (supplemented with compounds as described above) every other day for seven days when they were processed for muscle area determinations. All fly lines were maintained at 25° C on a standard day-night cycle. Climbing and flight assays were as described in^19^.

### Cross-sectional muscle area determination

Analysis of the IFM area in *Drosophila* thoraces was performed as previously described^48^. Briefly, six thoraces of seven-day-old females were embedded in Epon. Semi-thin 1.5 µm-sections were obtained using an ultramicrotome (Ultracut E, Reichert-Jung and Leica). Images were taken at 100 × magnification with a Leica DM2500 microscope (Leica Microsystems, Wetzlar, Germany). Five images containing IFMs per fly were converted into binary images using the ImageJ software, and the percentage of black pixels (corresponding to muscle) to the total number of pixels within a fixed-size frame was calculated.

### Cell culture and proliferation assay

Unaffected (control) and patient-derived (DM1) cells were kindly provided by Dr. Furling (Institute of Myology, Paris)^23^. Briefly, DM1 cells were obtained from a skin biopsy of an 11-year-old female donor expressing 1300 CTG repeats that have been immortalized and transduced to inducibly express MyoD (doxycycline). Control cells were obtained from a 25-years-old male non-DM1 donor. Cell lines are available at MYOBANK-AFM (Institute of Myology). Both donors, were unrelated. Forced expression of MyoD transdifferentiates fibroblasts into myotubes^23^” Fibroblast cells were grown in Dulbecco’s Modified Eagle’s Medium (DMEM) with 4.5 g/L glucose, 1% penicillin and streptomycin (P/S), and 10% fetal bovine serum (FBS; Sigma). To transdifferentiate fibroblasts into myotubes, the cells were plated in myotubes differentiation medium (MDM) containing DMEM with 4.5 g/L glucose, 1% P/S, 2% horse serum, 1% apo-transferrin (10 mg/ml), 0.1% insulin (10 mg/ml), and 0.02% doxycycline (10 mg/ml). In all cases, the cells were grown at 37 °C in a humidified atmosphere containing 5% CO_2_.

For toxicity assays, cells were seeded at 10^4^ cells/ml in 96-well plates differentiated for 96 h and treated with different concentrations of peptides (10 μM, 50 μM, 75 μM, and 100 μM); 48 h post-incubation, cell proliferation was measured using the CellTiter 96 AQueous Non-Radioactive Cell Proliferation Assay (Promega) following the manufacturer’s instructions. The IC_10_ and dose–response inhibition curves were calculated using non-linear least-squares regression, and absorbance levels were determined using a Tecan Infinite M200 PRO plate reader (Life Sciences).

The fusion index was defined as the percentage of nuclei within myotubes (> 2 myonuclei) regarding the total number of nuclei in each condition. The average number of total nuclei per myotube was determined by counting over 250 nuclei from randomly chosen Desmin-positive cells (5–7 micrographs).

### Immunofluorescence methods

For immunofluorescence detections, myotubes were fixed with 4% PFA for 15 min at room temperature (RT) followed by several washes in 1 × PBS. Cells were then permeabilized with PBS-T (0.3% Triton-X in PBS) and blocked (PBS-T, 0.5% BSA, 1% donkey serum) for 30 min at RT, and incubated with primary antibody mouse anti-MBNL1 (1:200, ab77017, Abcam), anti-Desmin (1:200, ab8470, Abcam) or anti-LC3 (1:2000, ab243506, Abcam) at 4 °C overnight. After several PBS-T washes, the cells were incubated for 1 h with a biotin-conjugated anti-mouse-IgG secondary antibody (1:200, Sigma-Aldrich) to detect anti-MBNL1, anti-Desmin, or anti-LC3. The fluorescence signal was amplified with an Elite ABC kit (VECTASTAIN) for 30 min at RT, followed by PBS-T washes and incubation with streptavidin-FITC (1:200, Vector) to detect anti-MBNL1 or anti-Desmin, for 45 min at RT. After several washes with PBS, the cells were mounted with a VECTASHIELD mounting medium containing DAPI (Vector) to detect the nuclei.

For MBNL1 and 79-biotin peptide co-localization assay, myotubes were fixed with 4% PFA for 15 min at RT followed by several washes in 1 × PBS. Cells were then permeabilized with PBS-T and blocked (PBS-T, 0.5% BSA, 1% donkey serum) for 30 min at RT and incubated with primary antibody mouse anti-MBNL1 (1:200, ab77017, Abcam) at 4 °C overnight. After several PBS-T washes, the cells were incubated for 1 h with a FITC-conjugated anti-mouse-IgG secondary antibody (1:200, Sigma-Aldrich) to detect anti-MBNL1. The fluorescence signal of the 79-biotin peptide was amplified with an Elite ABC kit (VECTASTAIN) for 30 min at RT, followed by PBS-T washes and incubation with streptavidin-TEXAS RED (1:200, Vector) for 45 min at RT. After several washes with PBS, the cells were mounted with a VECTASHIELD mounting medium containing DAPI (Vector).

For the detection of lysosomes, CNT and DM1 myotubes were treated as described above for immunofluorescence but incubated with 100 nM LysoTracker RED-DND99 and 5 µg/ml Hoechst 33258 (Invitrogen and Sigma-Aldrich, respectively) at 37 °C for 30 min, and were mounted using fluorescence mounting medium (Dako, Glostrup, Denmark). Images were taken at 400× magnification using a fluorescence microscope Leica DM4000 B LED.

For fluorescent in situ hybridization, fibroblasts were seeded into 96 well Cell Culture Plate (1 × 10^5^ cells per well) and treated with peptides. In situ detection was performed as previously described^49^. Images were taken and analyzed using an IN Cell Analyzer 2200 Imaging System (GE Healthcare).

### RNA extraction, RT-PCR, and quantitative real-time PCR (qRT-PCR)

Total RNA from human myotubes was isolated using TRIreagent (Sigma). One microgram of RNA was digested with DNase I (Invitrogen) and reverse-transcribed with SuperScript II (Invitrogen) using random hexanucleotides; cDNA was used in a standard PCR reaction with GoTaq polymerase (Promega). Specific primers were used to analyze the alternative splicing of *DMD, cTNT*, *SPTAN1*, *CAPZB*, and *DLG1* in control and DM1 human myotubes^12^. *GAPDH* was used as endogenous controls. PCR products were quantified using ImageJ software (NIH). For multiplex qRT-PCR we used 1 ng of human myotube cDNA as template using the QuantiFast Probe PCR Kit reagent. Commercial TaqMan probes (Qiagen) were used to detect human *MBNL1, MBNL2,* and *DMPK* expression levels with an Applied Biosystems StepOnePlus Real-Time PCR System. Results from myotubes were normalized to *GAPDH* (VIC-labelled probe; Integrated DNA Technologies). Expression relative to the endogenous gene and control group was calculated using the 2^−∆∆Ct^ method. Exon inclusion data come from at least three biological replicates, while real-time PCR was done with three technical replicates from each of three independent biological samples.

### Western blotting

Protein extracts were obtained from 1 × 10^6^ cells after sonication in RIPA buffer supplemented with protease and phosphatase inhibitor cocktails (Roche Applied Science). Total protein was determined by using Total protein concentration BCA protein assay kit.

(Pierce). For MBNL1 immunodetection 20 mg of total protein was heated 5 min at 100 ºC, electrophoresed on 12% SDS-PAGE gels, and transferred using a semi-dry system (Bio-Rad) onto nitrocellulose membranes (GE Healthcare). Then, membranes were blocked for 1 h at RT in PBS-T (0.05% Tween 20 [pH 7.4]) supplemented with 5% non-fat dried milk and incubated ON at 4 ºC with antibody mouse anti-MBNL1 (1:200, cloneMB1a, The Wolfson Centre for Inherited Neuromuscular Disease, UK) on blocking solution. After three washes with PBS-T membranes were incubated 1 h RT with secondary antibody in blocking solution (anti-mouse-IgG goat horseradish peroxidase (HRP)-conjugated 1:5000, Sigma-Aldrich). β-ACTIN was detected with a primary mouse anti-β-Actin antibody (1 h, 1:5000, Sigma-Aldrich) followed by HRP-conjugated anti-mouse-IgG antibody (1 h, 1:5000, Sigma-Aldrich). Bands were detected using ECL western blotting substrate (Pierce). Images were acquired using ImageQuant LAS 4000 (GE Healthcare).

### Differential scanning fluorimetry (DSF) and fluorescent indicator displacement (FID) assays

DSF experiments were performed to understand the peptides’ interaction with the double-stranded CUG RNA^38^. The experiment was performed using a StepOnePlus Real-Time PCR system (Life Technologies) with the melting curve software to measure the fluorescence intensity. A MicroAmp Fast Optical 96-well plate (Life Technologies) was used with 50 μl of solution per well. The RiboGreen dye was used at a final concentration of 300 nM, whereas the synthetic double-stranded CUG RNA (12 × CUG) was used at a final concentration of 600 nM. For each compound (in this case the four peptides and DMSO), four technical replicates were performed and, for each peptide, four different concentrations ranging from 1 to 100 μM were used. Sodium cacodylate buffer was used at pH 6.1, which is essential for experiments using RNA. During the DSF experiment, the temperature was increased from 4 to 95 °C at an increment of 0.2 °C with an equilibration time of 5 s at each temperature prior to measurement.

For each FID assays, a 1 ml solution containing the double-stranded CUG RNA (0.25 µM) and the indicator Thiazole Orange (0.75 µM) was prepared using cacodylate buffer (pH. 7.4). The solution was incubated at 25 ºC for 10 min before measuring the initial fluorescence spectrum. Then, aliquots of the tested compound (peptide 0.125 mM solutions in DMSO) were subsequently added up to saturation. The concentrations ranged from 0.03 up to 4 µM. After each addition, the cuvette was rigorously homogenized and let to stand for five min prior to measure the emission spectrum. The fitting procedure requires an asymptotical curve; therefore, a saturation final concentration is needed. All the experiments were performed at least in duplicate to ensure the reproducibility of the data. The values calculated for Ka and their associated errors come from averaging. The equipment used was a modular PTI fluorescence instrument (slit widths of 5 nm and power of 750 mV). The measurements were carried out using 1 ml quartz cuvettes with a path-length of 1 cm. The Thiazole Orange was excited at 495 nm and the emission spectrum was registered between 505 and 650 nm. The data was analyzed with the software HypSpec^50^. Once having established an initial equilibrium model for the interaction between the CUG, the TO and the peptide, the software applies an iterative algorithm in order to fit the experimental data to the proposed model, enabling the determination of the association constants^39,51^.

### Molecular modeling methods

The molecular structures of 79–82 peptides were created and prepared (capped with ACE and NME blocking groups and chirality modified) using the AMBER tleap module^52^. Molecular dockings were conducted using MOE 2019.01 software (Chemical Computing Group, Montreal, QC). The molecular structure of r(CUG)16 hairpin was previously modeled by homology^53^ using ModeRNA software^54^, and its stabilization was consequently studied through molecular dynamics simulations. Peptide structures were modeled in MOE and confronted with RNA, following the available induced fit protocol to consider both ligand and RNA as flexible structures. Triangle matcher algorithm was defined for placement, and binding energies were quantitatively estimated by GBVI/WSA dG rescoring function. The dynamic behavior of peptide-RNA complexes was assessed using molecular dynamics simulations using AMBER16 (University of California, San Francisco, CA). Molecular systems were prepared and solvated in TIP3P water solvent using tleap module. After solvent relaxation, constraining the RNA-peptide structure with a force constant of 2.0 kcal·mol^−1^ Å^−2^, the system was slowly heated at 300 K in 1 ns. A density equilibration stage under NPT ensemble (P = 1 bar) preceded the production stage, which was conducted at NVT conditions at 300 K during 10 ns. All simulations were performed under periodic boundary conditions, using the Particle Mesh Ewald (PME) to describe electrostatic interactions and SHAKE algorithm. The time step was fixed to 2 fs. Trajectory analysis, including the calculation of helical parameters, were conducted using cpptraj and considering the last 5 ns of the simulation^55^.

### Statistical methods

We assumed in all our experiments that parameters follow a normal distribution. In the molecular, functional, and histological analyses, pairs of samples were compared using two-tailed t-tests (α = 0.05), applying Welch’s correction when necessary. The statistical significance of the differences for all data reported can be found in Supplementary Table 2. Statistical analyses and plots were generated using GraphPad Prism. The sample size is stated in each figure. In the functional analysis of flight ability of the flies, Fisher's exact test was applied to compare percentages of flies able to fly with those unable.

## Supplementary Information

Supplementary Information.
