# Supplementary material for: Defined d-hexapeptides bind CUG repeats and rescue phenotypes of myotonic dystrophy myotubes in a Drosophila model of the disease
Source: Sci Rep. 2021 Sep 30;11:19417. doi: 10.1038/s41598-021-98866-0 (PMC8484449; doi:10.1038/s41598-021-98866-0)
Supplement: Supplementary file 1 — Supplementary Information. [file 41598_2021_98866_MOESM1_ESM.pdf]

Defined D-hexapeptides bind CUG repeats and rescue phenotypes of myotonic dystrophy myotubes in a *Drosophila* model of the disease

Anna Rapisarda, Ariadna Bargiela, Beatriz Llamusi, Isabel Pont, Roger Estrada-Tejedor, Enrique Garcia-España, Ruben Artero, Manuel Perez-Alonso

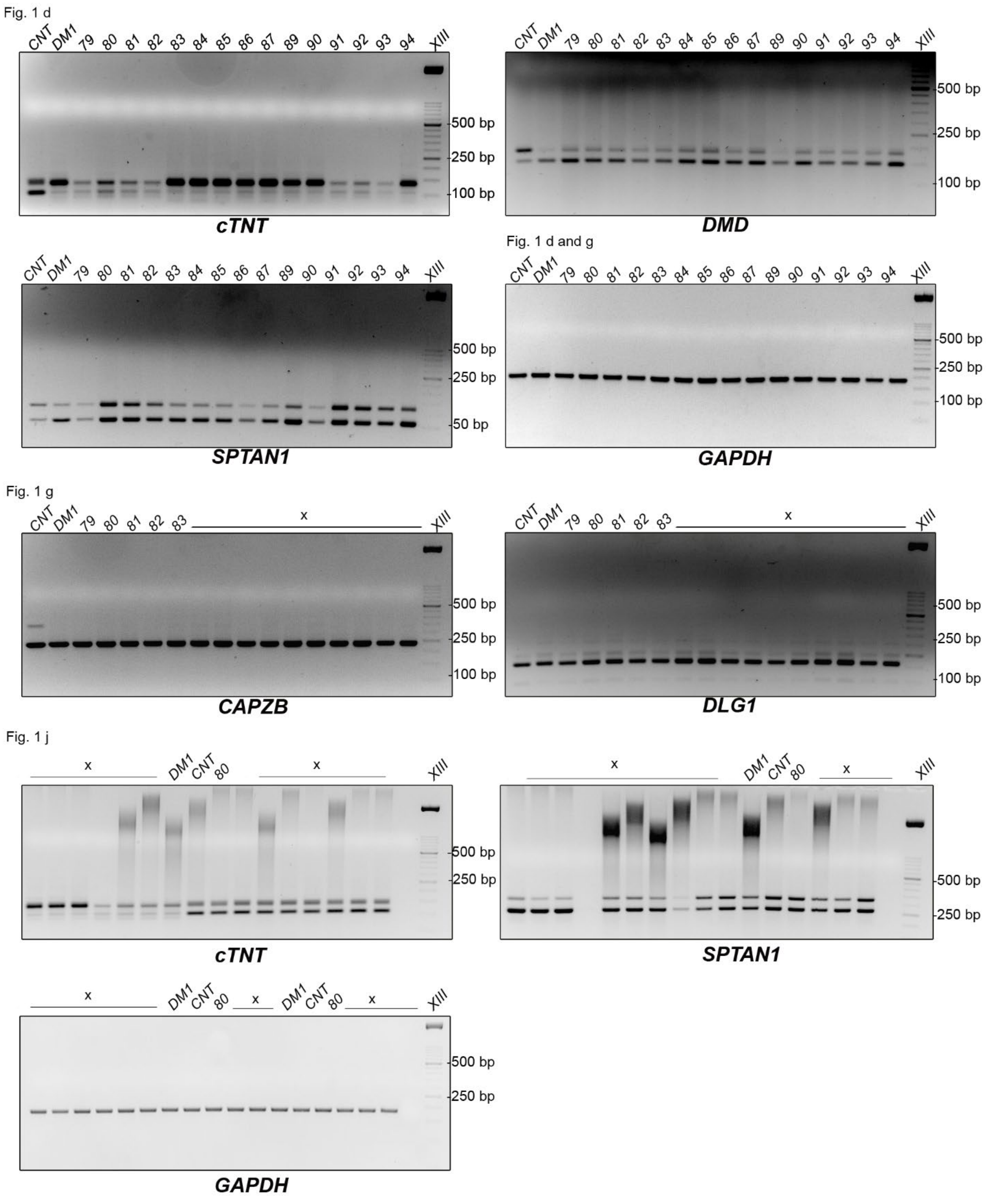

Supplementary Figure 1. *Uncropped agarose gels for figure 1. Crosses mark agarose gel lanes from unrelated experiments*

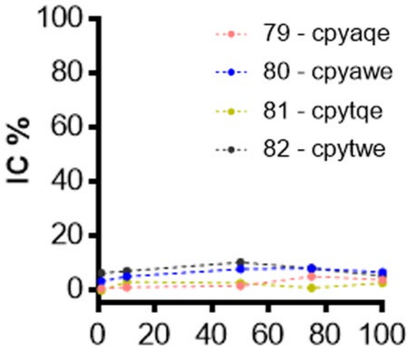

Supplementary Figure 2. Candidate peptides show no toxicity signs in fibroblasts*. Control fibroblasts were treated with the indicated concentrations of peptides (in µM) and the percentage of reduction in proliferation was obtained as described in the methods section.*

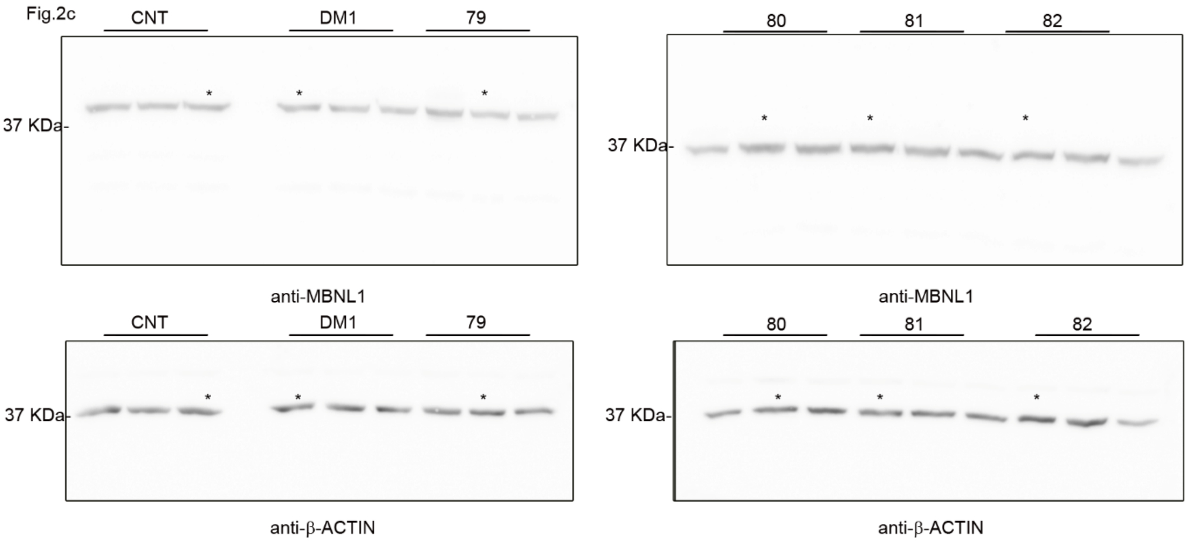

Supplementary Figure 3. Uncropped western blots for Figure 2. Biological replicates used to perform quantification are presented. Asterisks indicate representative lanes presented in Fig.2c

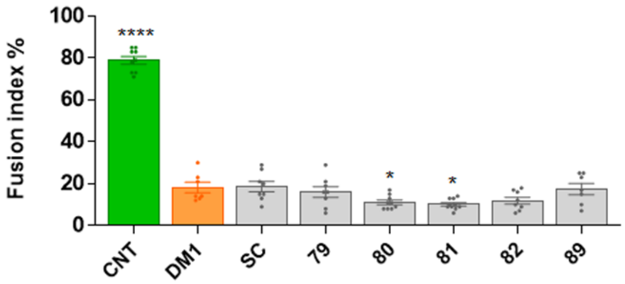

***Supplementary Figure 4**. **Candidate peptides do not rescue fusion index in disease myoblasts**. Fusion index of normal (CNT) and DM1 myoblasts treated with vehicle only (DMSO; DM1) or the indicated peptides (SC, scrambled negative control). Individual data points are represented. Statistically significant differences are denoted according to a Student´s t-test; * p<0.05.*

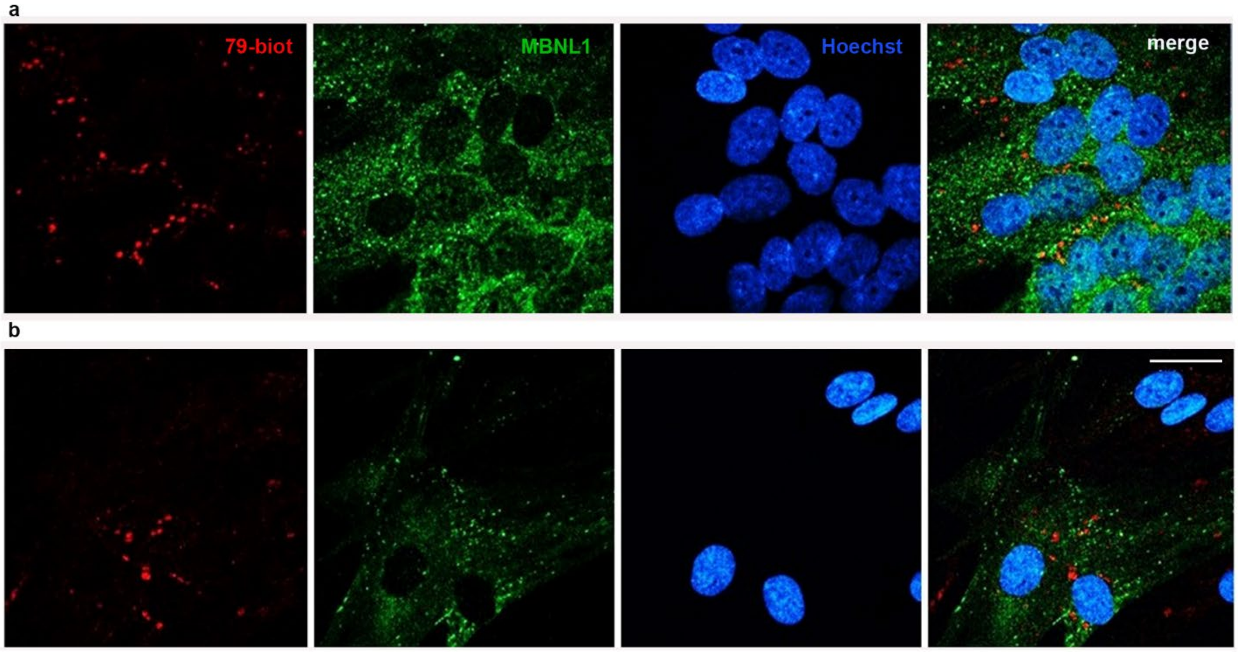

***Supplementary Figure 5**. **Peptide 79 does not significantly overlap with the MBNL1 protein intracellularly.** Double immunostaining with biotin-labeled peptide 79 (red channel) and anti-MBNL1 (green channel) revealed that the peptide accumulated in the cytoplasm of DM1*

myoblasts, mainly in the perinuclear area (blue nuclei; Hoechst stained), with no overlap with MBNL1 (merge panels). Scale bar corresponds to 20 microns.

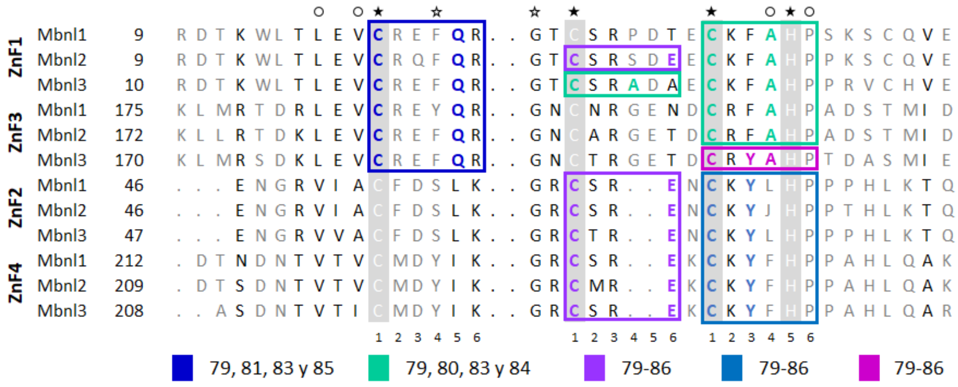

Supplementary Figure 6. Sequence identities between candidate peptide sequences and zinc fingers of MBNL1, 2 and 3.

Supplementary Table 1. The sequence of all D-amino acid hexapeptides used in this work.

Peptide	Sequence
79	Ac-cpyaqe-NH_2
80	Ac-cpyawe-NH_2
81	Ac-cpytqe-NH_2
82	Ac-cpytwe-NH_2
83	Ac-cqyaqe-NH_2
84	Ac-cqyawe-NH_2
85	Ac-cqytqe-NH_2
86	Ac-cqytwe-NH_2
87	Ac-ppyaqe-NH_2
88	Ac-ppyawe-NH_2
89	Ac-ppytqe-NH_2
90	Ac-ppytwe-NH_2
91	Ac-pqyaqe-NH_2
92	Ac-pqyawe-NH_2
93	Ac-pqytqe-NH_2
94	Ac-pqytwe-NH_2
scramble	Ac-pypaew-NH_2

Supplementary Table 2. P values for all the statistical comparisons indicated according to Student´s t-tests.

Figure	Panel	Column	p-value
1	a	CNT	0.0010
		79	0.0282
		80	0.0026
		81	0.0075
		82	0.0010
		91	0.0132
		92	0.0042
	b	CNT	<0.0001
		79	0.0351
		80	0.0004
		81	0.0235
		82	0.0154
		83	0.0002
		84	<0.0001
.		85	0.0003
		87	0.0219
		90	0.0013
		91	0.04
		92	0.0271
		93	0.0002
	c	CNT	< 0.0001
		79	0.0074
		80	0.0002
		81	0.0003
		82	0.0003
		83	0.0189
		84	0.0031
		85	0.0047
		91	0.0015
		92	0.0024
	e	CNT	< 0.0001
	h	CNT	< 0.0001
		80	< 0.0001
	i	CNT	0.0033
2	a	79	< 0.0001
		80	0.0001
		81	< 0.0001
		82	0.0005
	b	79	0.0137
		81	0.0003
	c	CNT	0.0363
		80	0.0091
		81	0.0013
3	i	CNT	< 0.0001
		79	0.0016
		80	0.385
		81	0.0198
	j	CNT	< 0.0001
		79	< 0.0001
		80	0.0010

		81	0.0018
		82	0.0006
4	i	CNT	< 0.0001
		79	0.0277
		81	0.0003
5	a	CNT	0.0024
		79	0.0220
		80	0.0058
		81	0.0011
		82	0.0007
	j	CNT	0.0030
		89	0.0015
		sc	0.0019
		79	< 0.0001
		80	< 0.0001
		81	< 0.0001
		82	< 0.0001
	k	CNT	< 0.0001
		79	0.0223
